# Human-Computer Interaction Based on Hand Gestures Using RGB-D Sensors

**DOI:** 10.3390/s130911842

**Published:** 2013-09-06

**Authors:** José Manuel Palacios, Carlos Sagüés, Eduardo Montijano, Sergio Llorente

**Affiliations:** 1 Departamento de Informática e Ingeniería de Sistemas (DIIS) and Instituto de Investigación en Ingeniería de Aragón (I3A), Universidad de Zaragoza, Zaragoza 50018, Spain; E-Mail: csagues@unizar.es; 2 Centro Universitario de la Defensa (CUD) and Instituto de Investigación en Ingeniería de Aragón (I3A), Zaragoza 50090, Spain; E-Mail: emonti@unizar.es; 3 Research and Development Department, Induction Technology, Product Division Cookers, BSH Home Appliances Group, Zaragoza 50016, Spain; E-Mail: sergio.llorente@bshg.com

**Keywords:** Kinect, depth sensors, RGB-D, gesture recognition

## Abstract

In this paper we present a new method for hand gesture recognition based on an RGB-D sensor. The proposed approach takes advantage of depth information to cope with the most common problems of traditional video-based hand segmentation methods: cluttered backgrounds and occlusions. The algorithm also uses colour and semantic information to accurately identify any number of hands present in the image. Ten different static hand gestures are recognised, including all different combinations of spread fingers. Additionally, movements of an open hand are followed and 6 dynamic gestures are identified. The main advantage of our approach is the freedom of the user's hands to be at any position of the image without the need of wearing any specific clothing or additional devices. Besides, the whole method can be executed without any initial training or calibration. Experiments carried out with different users and in different environments prove the accuracy and robustness of the method which, additionally, can be run in real-time.

## 1. Introduction

In recent years, hand gesture recognition is gaining great importance in human-computer interaction (HCI) and human-robot interaction (HRI). Different approaches have appeared making use of different sensors and devices. Hand wearable devices such as sensor gloves [1,2] have been used although they are usually expensive and user intrusive. Other less intrusive wireless devices like the Wii controller [3] or sensing rings [4] have appeared to overcome these drawbacks. Cameras and computer vision have proved to be useful tools for this task [5]. In addition, other contact-free sensors have emerged lately [6] to detect hand motion and interact with different devices. However, despite all the previous work, a reasonable solution to the gesture recognition problem has not been found yet.

Modern approaches to gesture recognition usually acquire information from the lately developed colour and depth-sensing devices. The first colour and depth sensor called Kinect was developed by Microsoft for the Xbox console and released in November 2010. This sensor projects an infrared pattern of 307,200 dots in a 640 × 480 mesh and receives the reflected pattern through a CMOS monochrome sensor. This structured light application allows the device to measure the depth of every point by means of triangulation. Moreover, an RGB camera provides synchronised colour information of each point.

Microsoft Kinect was formerly developed for full-body tracking to interact with video games by means of body movements and gestures. This sensor and its followers have proved to be suitable for such task and many body-tracking methods have appeared lately. Furthermore, many authors have developed applications of gesture recognition using these sensors in different fields such as interactive displays [7], physical rehabilitation [8], robot guidance [9–11] or sign language recognition [12]. Additionally, completely different applications for Kinect have been developed such as [13,14].

Nevertheless, even with the appearance of these new sensors, finding and segmenting the hand of the user in an image is still a meaningful problem. It remains unsolved especially in situations where there are occlusions, different lighting conditions or when other skin-coloured objects apart from the hand appear in the scene [15]. In the last years, hand gesture recognition applications have focussed on the recognition problem itself, simplifying the problem of finding the user's hand. Common simplifications are the assumption of some particular situations like the hand being the front-most object [16] or the use of full-body tracking algorithms. Under these assumptions, different gesture classification methods such as Hidden Markov Models [12,17], k-Nearest Neighbours [10], Template Matching [16] or Finite State Machines [9] have reached high classification rates.

In this paper, we propose a new method to detect the hands and recognize its static and dynamic gestures. A colour and depth sensor is used, particularly Microsoft Asus Xtion Pro Live. One of the main goals of our work is to provide the user with freedom of movement. Our method allows: the appearance of the hand in any position; the user wearing short-sleeved shirts and no additional items; the appearance of the user's face; and the use of the system in presence of cluttered backgrounds.

In order to recognize hand gestures, the hand pixels have to be identified. Hand regions are smaller than 64 × 64 pixels when the user stands approximately 3 meters away from the sensor. To cope with this weakness of low resolution of the sensor in the hand region, we assume that the user is frontal to the camera and less than 2 meters away from it so that we can perform a simple depth threshold. In comparison with traditional video-based hand gesture recognition approaches, this assumption solves the repeatedly faced problem of cluttered background. In addition, the distance of use that appears in the sensor specifications starts at 0.8 m. Thus, the user should not hold his hand nearer than this distance since the sensor does not return any measurement. In this work, we address the problem of differentiating the hand between all the skin regions segmented by a colour filter. First, we find the faces and the hands of the users, if they appear. Afterwards, we determine the position of the palm and the wrist to accurately separate the hand from the forearm. This achievement lets the user's arm be within view and provide considerably more freedom in reference to pose and clothes. With the information of the hand, we present a new gesture recognition approach based on a feature-based decision tree. Apart from static gestures, we also develop a simple dynamic gesture recognition system to identify linear movements of the hand. The different steps followed by our system are shown in Algorithm 1. Finally, we carry out several experiments to test its performance and attach a video that shows the real time static and dynamic gesture recognition.


**Algorithm 1**
*Overview of the whole system*
1:*Hand segmentation*2:*Feature extraction*3:*Static gesture classification*4:*Dynamic gesture classification*


In contrast with other works, our principal contributions are: (1) We distinguish faces from hands, letting the user hold any pose in front of the camera; (2) The user's hands can appear anywhere in the scene not necessarily being the front-most object; (3) We identify the wrist so as to separate the forearm making the system sleeve-length independent; (4) Any number of hands and faces can be shown at the same time; (5) Neither training nor calibration pose are required. In order to allow (2) and (3), we present two novel methods. The first one distinguishes the circle associated to the palm with the maximum circle that can appear in the elbow region. The second one finds the forearm with respect to the hand. Additionally, we introduce a new approach to static gesture classification using a feature-based decision tree. Finally, we propose a novel dynamic gesture recognition method to determine the directions of movement of a spread hand.

The remainder of this paper is structured as follows: Section 2 presents the procedure to segment the hands from the scene, Section 3 describes the algorithm for static gesture classification, in Section 4 dynamic gesture recognition is introduced, Section 5 describes the experiments carried out to test the system and Section 6 draws the conclusions of this work.

## 2. Hand Segmentation

Traditional vision-based hand segmentation methods are commonly based on colour filtering. These methods are seriously affected by the appearance of skin colour-like objects and by lighting conditions. More recent approaches to hand gesture recognition with depth sensors have in some way forgotten the availability of synchronised RGB information. However, some authors [18,19] combine colour and depth information to obtain a satisfactory hand segmentation for their work. The coexistence of both types of data allows to take advantage of their strengths at the same time.

In the following, we describe our approach to segment the hands present in the image, which is divided in three steps. In the first one a series of different filters is applied to remove all the parts of the image that do not correspond to human skin regions. The second step classifies the different blobs into different human body parts, discerning between faces and arms. The last part of the algorithm extracts the whole hand and its position with respect to the forearm. The whole scheme is summarized in Algorithm 2.


**Figure 1.**
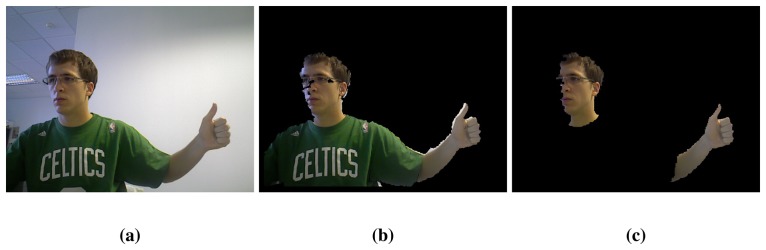
Skin region segmentation: (**a**) Original image; (**b**) Background subtraction; (**c**) Skin color segmentation.

**Figure 2.**
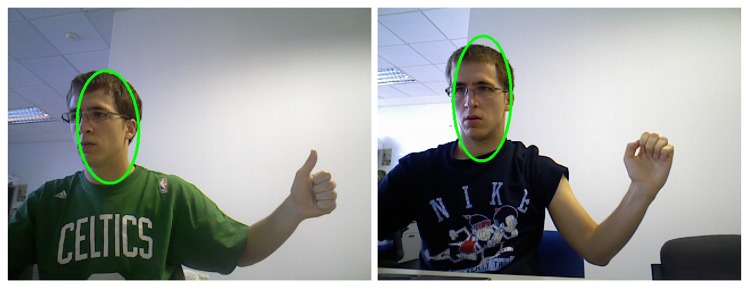
Skin region classification: Detection of blobs that correspond to human faces.

**Figure 3.**
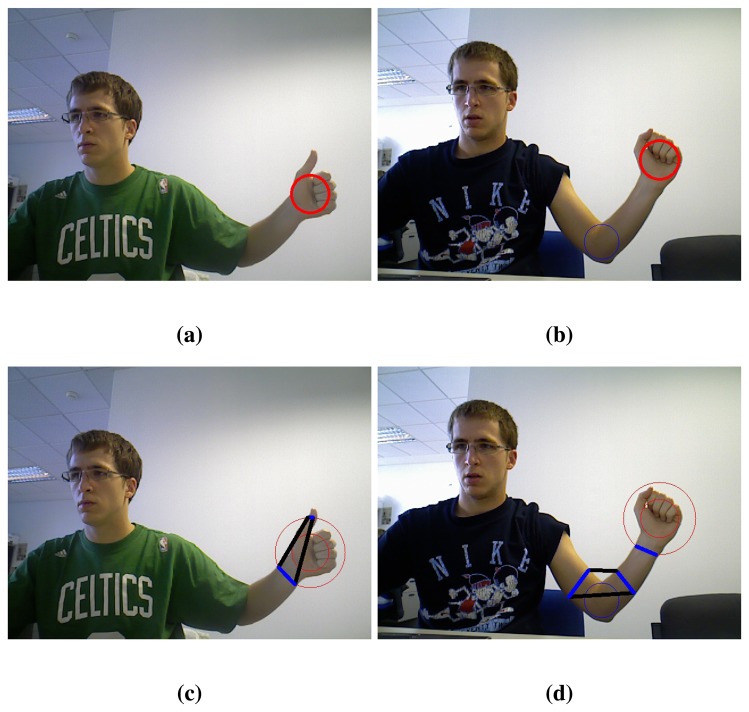
Skin region classification: (**a**) Palm circle when the elbow is not shown; (**b**) Palm and elbow circles; (**c**) Skin segments (blue lines) of the hand; (**d**) Skin segments (blue lines) of hand and elbow.

**Figure 4.**
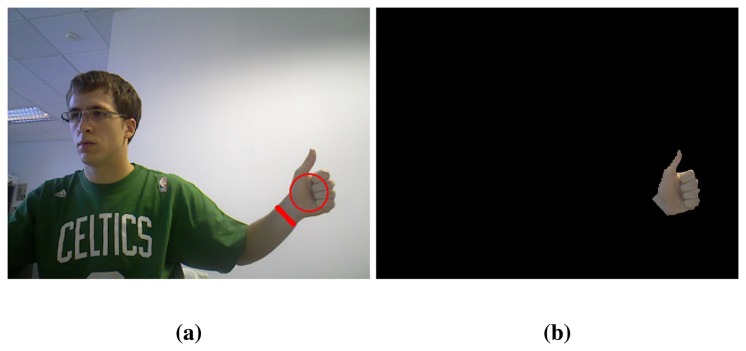
Hand extraction: (**a**) Palm circle and wrist segment identification; (**b**) Hand extraction.

**Algorithm 2**
*Hand Segmentation*
**Require:** Input image ℐ (Figure 1a)1:– *Skin region segmentation*2: Depth filter (Figure 1b)3: Colour filter (Figure 1c)4:– *Skin region classification*5: Detect and remove faces (Figure 2)6: Classify the extremes of the arm (Figure 3)7:– *Hand extraction*8: Detect the wrist (Figure 4a)9: Extract the hand (Figure 4b)


## 2.1. Skin Regions Segmentation

Some regions clearly do not represent human hands either because of their position in the image or because of their appearance. The first part of the algorithm is oriented to the removal of all these non-essential parts of the image. Initially, a depth threshold is performed to the original RGB-D image. This eliminates all sources of confusion related to the background (Figure 1a,1b). This filter also solves the problem of cluttered backgrounds, which strongly affects skin colour segmentation methods and preserves enough resolution for hand regions. At this point, the advantage of using 3-dimensional information justifies the use of an RGB-D sensor.

Once the background has been removed from the image we use a colour filter to remove all the parts that do not represent human skin like t-shirts or other clothing. Skin colour detection techniques have been very useful as a preliminary step in HCI applications thanks to the consistency of skin colour and their computational effectiveness. Among the different methods that can be used to model the colour of the human skin [20], we have opted for defining an explicit skin colour cluster to distinguish the skin regions. The advantages of following this approach are that it does not require a training stage, it is less complex and has been more frequently used.

The HSV space makes colour hue and saturation independent from illumination. Therefore, the skin tone region can be delimited in the lighting-independent H-S plane by setting suitable thresholds:

$$\begin{array}{c} V>40\\ 25<S<153\\ H<22\;\text{or}\;H>168 \end{array}$$

Since small spurious objects can appear in some images due to clothes patterns, all objects with a small area are removed. In addition, skin regions can show defects and holes as a result of the colour filtering. The closing morphological operation fills the holes and smooths contours. Figure 1c illustrates this process.

Once all background and non-skin objects are eliminated from the scene, it is perfectly reasonable to assume that the only remaining objects will be hands, arms and faces of different users considering that they can overlap. In such cases, if the depth histogram of any skin cluster shows two separated regions, another depth threshold is performed whereby the overlapped region of the back of each object is eliminated. This simple operation overcomes the problem of superimposed hands and faces that appear as the same object after the colour filtering.

### 2.2. Skin Regions Classification

Among the already non-overlapped skin regions, faces are the easiest to classify since they can be modelled as rigid objects for this purpose. Many face detection algorithms have been proposed using Neural Networks [21], machine learning techniques [22] or even adapted techniques to be used with the Kinect sensor [23]. In this work, the approach proposed in [24] is followed since it is simple, fast and robust. According to [24], faces present two features that allow them to be differentiated from hands: their approximately elliptical shape and their orientation. Three tests are carried out to decide if an object is a face or not. We calculate the best-fit-ellipse of the contour of each skin blob in terms of the least square error. We compute its orientation with respect to the vertical axis, its aspect ratio and the relation of areas between this ellipse and the original blob. These values determine whether the contour represents a face. Figure 2 shows in green the ellipses of the blobs detected as faces.

Once faces have been recognised, hands may appear alone due to long sleeves or together with arm and forearm. In some cases, the hand can be the front-most part of the cluster but, in general, it cannot be separated from the arm only with depth information. Hence, the palm is found as the maximum inscribed circle that fits in the hand contour (Figure 3a). However, this circle can appear in the elbow when the user is wearing a short-sleeved shirt. To avoid this, two different circles are found, the maximum inscribed circles with centre in each half of the blob (Figure 3b).

At this point, the skin segments are computed. At first, intersections between the hand contour and a circumference concentric to the palm circle are extracted. Skin segments are the segments between two consecutive intersections that run over a skin region (Figure 3c, 3d).

The palm is selected between the two circles, namely A and B, by their number of skin segments using a look-up table (Table 1). Due to the arm anatomy, in the hand region one to six skin segments can appear whereas in the elbow region only one or two. In the vast majority of cases two and only two segments appear in the elbow zone if a circle proportional to the one of the palm has been detected. Therefore, further comparison is needed only when there are two segments in each region. In that case, the sum of the segments' longitude must be greater in the elbow region. In Figures 3b and 3d an example of the palm circle selection can be seen.

### 2.3. Hand Extraction

The palm circle determines the hand position but, nevertheless, the position of the forearm relative to the hand remains uncertain. In addition to the circumference mentioned before, called *first circum-ference*, skin segments are computed for a bigger circumference (*second circumference*). Let us denote *N Seg*1 and *N Seg*2 as the number of segments found in the *first* and *second circumference* respectively. With this information, plus the length of the different segments, the wrist can be found following a simple search tree algorithm that performs the comparisons shown in Algorithm 3. The last part of the algorithm considers the aspect ratio of the blob to determine whether it contains an arm or only a hand. A sufficiently small aspect ratio of the blob, *i.e.*, similar to a circle, implies that there is only a hand. A big aspect ratio, *i.e.*, an ellipse with dissimilar axes, indicates the presence of the arm.


**Algorithm 3**
*Arm and Wrist Detection*
**Require:**
*N Seg*1, *N Seg*2 and the length of the segments1:*N Seg*1 = 0 ⇒ No Arm2:*N Seg*1 = 1 ⇒3: *N Seg*2 = 0 ⇒ No Arm4: *N Seg*2 = 1 ⇒5:  Length *Seg*1 > Length *Seg*2 ⇒ No Arm6:  Length *Seg*1 ≤ Length *Seg*2 ⇒ Assign wrist segment7: *N Seg*2 ≥ 2 ⇒ No Arm8:*N Seg*1 ≥ 2 ⇒9: Small aspect ratio of the blob ⇒ No Arm10: Big aspect ratio of the blob ⇒ Assign wrist segment


The selected wrist segment, in those situations where there is one (Figure 4a), allows us to eliminate the forearm. All the points of the blob that are on the opposite side of the wrist segment with respect to the palm centre are removed. Thereby, the user hand is definitely obtained (Figure 4b).

This approach let the user's arm be within view providing considerably more freedom in reference to pose and clothes. In fact, the method of extracting the hands presented in this paper imposes no restriction, neither to movements nor to objects that appear in the scene. This method distinguishes the hand whatever its position is, even in presence of cluttered backgrounds. Additionally, this procedure can handle any number of hands and faces including the possibility of overlapping.

## 3. Hand Gesture Recognition

As mentioned in [15] there are two main approaches to gesture classification: machine learning algorithms or feature-based classifiers. We present a classifier of the second type, making use of the features extracted in the previous section to differentiate the hand from the rest of the arm plus other additional features. In particular, besides the palm centre, the number of fingertips and its locations are found so as to classify gestures. The classification process is schematised in Algorithm 4.


**Figure 5.**
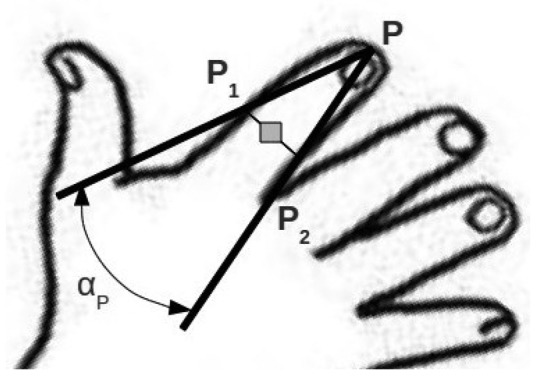
Curvature calculation.

**Figure 6.**
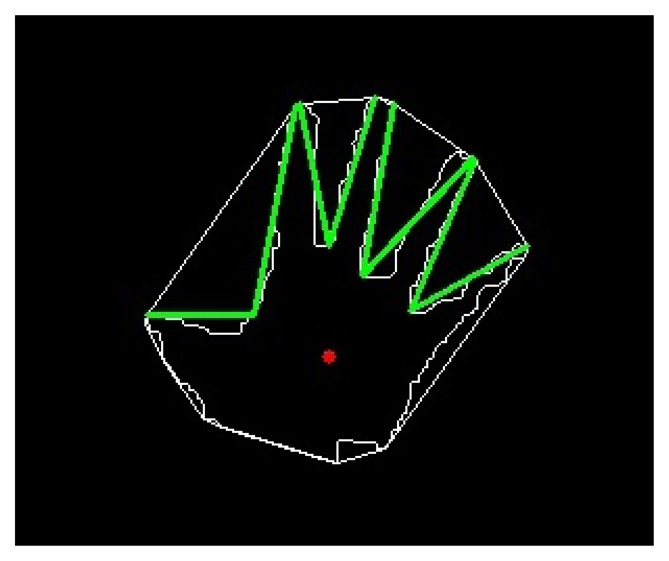
Convexity defects.

**Figure 7.**
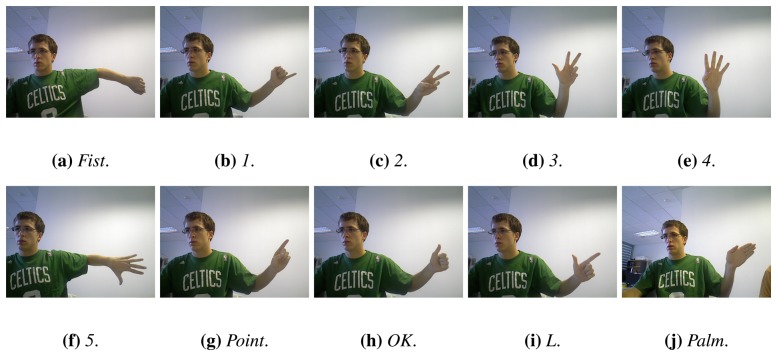
Examples of the ten gestures available in the lexicon.

**Algorithm 4**
*Hand Gesture Recognition*
**Require:** Image of the extracted hand (Figure 4b) and a vocabulary of hand gestures (Figure 7)1:– *Fingertip detection*2: Compute the maximums of curvature (Figure 5)3: Compute the defects of convexity (Figure 6)4:– *Gesture classification*5: Recognize the gesture (Algorithm 5)


### 3.1. Fingertip Detection

The first feature we extract from the hand to identify the number of fingertips is the number of maximums of curvature of the hand contour. The curvature of each contour point can be defined as follows:

$$K_{P}=\cos(\alpha_{P})=\frac{\vec{P_{1}P}\cdot \vec{PP_{2}}}{\left\|\vec{P_{1}P}\right\|\cdot \left\|\vec{PP_{2}}\right\|}$$

where P is the contour point and *P*_1_ and *P*_2_ are contour points at a distance of *l* contour points at each side of P. (Figure 5).

Due to the long and narrow shape of fingers, local maximums of curvature in the contour of the hand are reached at fingertips and at the middle point between two separated fingers. Both types can be separated by checking if the segment $\vec{P_{1}P_{2}}$ runs through the skin blob, which holds for fingertips. This differentiation provides additional information to determine whether the fingers are together or separated. With the location of the fingertip the finger direction is determined by the vector $\vec{QP}$, where Q is the middle point of the segment $\vec{P_{1}P_{2}}$.

Other valuable features are the convexity defects, which measure the deviation of the contour with respect to its convex hull. A defect is defined as the space between two points that lie both on the convex hull and in the hand contour. It is unambiguously delimited with four parameters: initial and final points, maximum depth and its corresponding position. The depth of the defect between two fingers can be seen as measurement of the finger's length. As this measurement depends on how far the hand is from the sensor, we normalise it by the palm radius to make it scale-independent. This normalised depth is sufficient to conclude whether the defect belongs to a space between two fingers, to a spurious contour or if it does not belong to a spread finger. In Figure 6 we show the convexity defects associated with fingers.

Separately any of these two features contains enough information to determine the number of spread and separated fingers. Nevertheless, in order to make the recognition more robust, we use a combination of both features, checking the consistency of both to make a decision about the total number of fingertips.

### 3.2. Gesture Definition

Once the features have been extracted, a description of the gestures that the system must classify is needed. We set a 10 gesture lexicon that includes from 0 to 5 spread and separated fingers and 4 common gestures: *palm*, *OK*, *L* and *point*. Examples of these gestures are given in Figure 7. It is worth mentioning that three out of these four gestures are particular cases of a number of spread fingers. *Point* and *OK* are particular cases of one finger and *L*, such of two. In addition, this lexicon includes all the possible combinations of spread fingers that means a wide variety of gestures.

### 3.3. Gesture Classification

With the definitions of the gestures and the features previously obtained, a feature-based decision tree is applied to classify the gesture that is being held. This classification is summarised in Algorithm 5. The algorithm uses the information about the number of segments and the lengths obtained in Section 2.3 besides the number of fingertips, computed in Section 3.1. The number of segments found in the concentric circumferences around the palm centre allows us to discern *palm* and *fist gestures*. For the rest of the gestures we use the number of spread and separated fingertips. However, the particular *OK*, *Point* and *L gestures* remain unnoticed. For this purpose two different situations are distinguished:

1. One spread finger: the distance from the fingertip to the palm centre and the angle to the forearm, if it is known, are measured. On the one hand, thumb, *i.e.*, *OK gesture*, has the shortest distance and its direction makes the smallest angle with the forearm. On the other hand, any of the other fingertips, *i.e.*, *Point gesture*, is at a considerable longer distance and makes a bigger angle.
2. Two spread and separated fingers: in this case, when *L gesture* is being held, the directions of the two fingers, *i.e.*, thumb and another one, set a bigger angle than other two fingers holding a *2 gesture* and not including thumb.

**Algorithm 5**
*Gesture Classification*
**Require:**
*N Seg*1, *N Seg*2 with their lengths and the number of fingertips (*N_F_*)1:– *Palm*2: *N Seg*1 = *N Seg*2 = 1 and Length *Seg*1 > Length *Seg*23: *N_F_* =04:– *Fist*5: *N Seg*1 = 06: *N Seg*1 = *N Seg*2 = 1 and Length *Seg*1 ≤ Length *Seg*27: *N_F_* =08:– *OK*9: *N Seg*1 = 1 and *N Seg*2 = 0 and *N_F_* = 110:– *Fingers* 1,…, 511: *N Seg*1 ≥ 2 and the corresponding value of *N_F_*12: – *Point*, *OK and L*13:   Special cases of one and two fingers (check length and angle;


## 4. Dynamic Gesture Recognition

For several HCI or HRI applications, the recognition of natural and simple dynamic gestures becomes of quite interest for tasks like menu navigation or robot guidance. For that reason we extend our method to easily recognize motion of the static *5 gesture*. This gesture is the most natural and intuitive gesture that humans use in daily life, usually to say hello, or even when they are unexpectedly in front of a camera. Six movements corresponding to positive and negative directions on the coordinate axes are considered. Thereby, the lexicon of dynamic gestures consists of *up*, *down*, *right*, *left*, *forwards* and *backwards*. Algorithm 6 shows a schematic view of this process.


**Algorithm 6**
*Dynamic Hand Gesture Recognition*
**Require:** 3D position of gesture *5* along several frames1:– *Dynamic Gesture Classification*2: Compute distance and direction of motion of the hand (Equations (1)–(3))3: Identify the dynamic gesture (Figure 9)


As our goal is solely to determine these linear motions of *5 gesture*, hand tracking consist in storing the 3-dimensional position of the palm centre of the hand that is holding this gesture. For every frame of a video sequence in which a *5 gesture* appears, its position is stored. If a near position has been previously stored, the new position is appended. This means that the hand is making a gesture. If, on the contrary, it is the first time that such gesture appears in its proximity, it becomes the first stored position.

**Figure 8.**
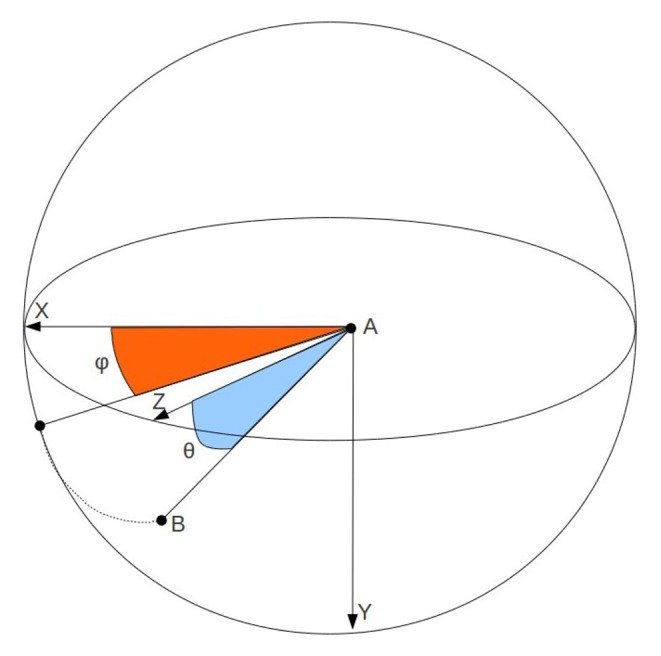
Trajectory angles.

With the last n positions of a hand, its trajectory is computed to decide whether the user is performing a dynamic gesture. Trajectory is represented as follows:

- Distance: the Euclidean distance between the first and last stored positions.
  (1) $$d=\sqrt{{\left(x_{n}-x_{0}\right)}^{2}+{\left(y_{n}-y_{0}\right)}^{2}+{\left(z_{n}-z_{0}\right)}^{2}}$$
- Direction: denoted by the pair of angles of the spherical coordinates (Figure 8).

The direction of the trajectory is obtained by the mean of the angles between each pair of stored positions:

(2) $$\bar{\varphi }=\frac{\Sigma \varphi_{i}}{n}\;\bar{\theta }=\frac{\Sigma \theta_{i}}{n}$$

However, these values do not clearly represent the hand trajectory if the hand has changed its direction of movement in the middle of the gesture. Therefore, the standard deviation of both angles is necessary:

(3) $$\sigma_{\varphi }=\sqrt{\frac{\Sigma {\left(\varphi_{i}-\bar{\varphi }\right)}^{2}}{n-1}}\;\sigma_{\theta }=\sqrt{\frac{\Sigma {\left(\theta_{i}-\bar{\theta }\right)}^{2}}{n-1}}$$

**Figure 9.**
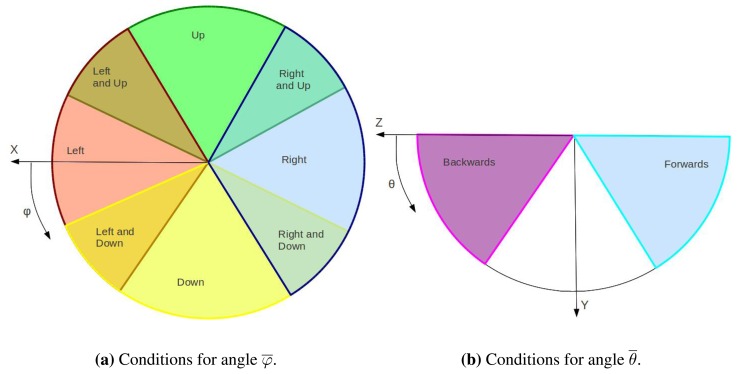
Dynamic hand gestures classification.

A low value of these deviations means that the movement of the hand is monotonous and rectilinear. If such circumstance arises and the distance value is high enough, the user has made a dynamic gesture whose direction is represented by *φ̄* and *θ̄*. In that case, the gesture is classified according to Figure 9.

## 5. Results and Discussion

To test the efficiency and the robustness of our static gesture recognition system, we carried out a series of experiments. We took a total of 90 images of 9 different subjects in which each subject represented all the static gestures. These images were taken in different environments to check the behaviour in different lighting conditions and with different objects appearing in the scene. Moreover, the experiments were carried out according to the following statements:

- Users received a brief explanation of the operation of the system and the gesture lexicon.
- Users enjoyed total freedom to choose the gesture, its position and the way of making it.
- No restrictions in terms of clothing and sleeve length were imposed except from skin colour-like clothes.

Before carrying out these tests, we selected the parameters of the system using a data set consisting of 60 images of 3 different users:

- The thresholds obtained for face detection are shown in Table 2 in comparison with [24]. Differences mainly lay in the inclusion of the user's neck in the face blob. Such part enlarges the ellipse allowing a bigger aspect ratio and reducing the orientation value. A lower limit in the relation of areas is required due to some pixels of the face border that are not correctly classified as skin.
- According to the hand anatomy, a circumference of 1.57 times the palm circle radius intersects with all fingers. In contrast, we choose a value of 1.85 to find skin segments as a result of the dissimilarities between a real hand and the detected hand. In addition, when finding the skin segments with a bigger circumference, its radius is chosen 2.6 times the radius of the palm circle.
- A maximum of curvature is considered to be a fingertip if its value is bigger than 0.8, which represents approximately *α*_P_ < 36°. Moreover, convexity defects are classified as follows:
  1. Any defect with a bigger normalised depth than 0.7 belongs to a space between two fingers.
  2. Any defect with a bigger normalised depth than 2 belongs to an spurious contour.
  3. Any defects with a normalised depth between 0.2 and 0.7 does not belong to a spread finger.
  4. Common gesture like a fist or a palm with all fingers spread and together can have no defect deeper than 0.2 times the palm radius.
- In order to correctly classify particular gestures, the following rules are applied:
  1. Thumb tips, *i.e.*, *OK* gestures, are nearer than 2.5 times the palm radius and its directions are at an angle smaller than 140° to the forearm. Any other fingertip, *i.e.*, *point gesture*, is between 2.5 and 3.5 times the palm radius from the palm centre and its angle is bigger than 140°.
  2. *L* gesture is being held if the angle between both fingers is bigger than 45°. In other case, it is gesture *2*.

We evaluated the performance of our system using two different parameters frequently used in computer vision:

$$\text{precision}=\frac{tp}{tp+fp}\cdot 100\;\text{recall}=\frac{tp}{tp+fn}\cdot 100$$

where *tp* is the number of true positives; *fp*, the number of false positives; and *fn*, the number of false negatives.

The global results are shown in Table 3. Our system reached an overall *precision* of 92.1% whereas *recall* was 83.3%. These values are satisfactory taking into account that no constraints are imposed to the user who can show his face, forearm and arm and locate his hand anywhere in the scene.

**Figure 10.**
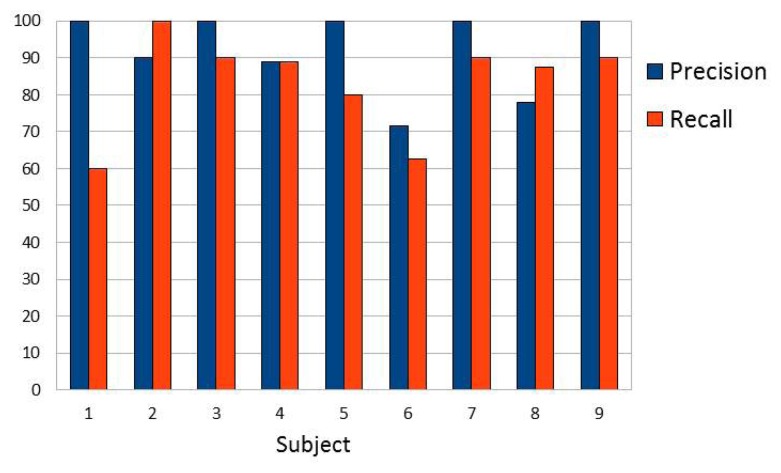
Statistics for different subjects.

**Figure 11.**
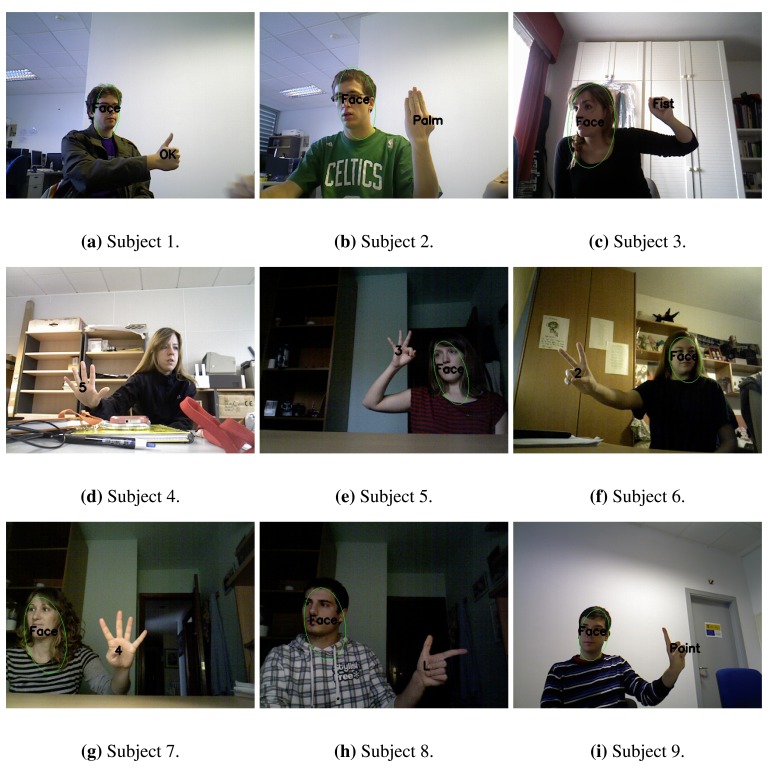
Images of the different users.

Results can also be analysed individually for each subject. In Figure 10 *precision* and *recall* are presented and it can be extracted that the biggest (subject 1) and the smallest hands (subject 6) achieve less *recall*. In Figure 11 one example of each user is shown. These examples show how the system works with different lighting conditions (natural, artificial or even low light), different skin tones of the users, cluttered backgrounds, skin colour-like objects, different sleeve lengths and clothes and different hand positions.

**Figure 12.**
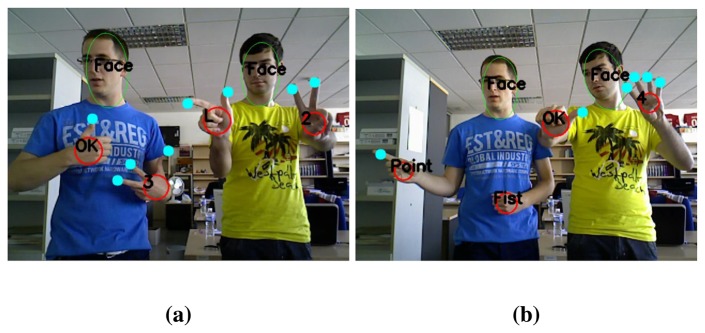
Examples of the appearance of two different users of the system simultaneously.

In Figure 12, two different users appear in front of the sensor at the same time. These images demonstrate that the proposed method is capable of handling several hands and faces simultaneously.

**Figure 13.**
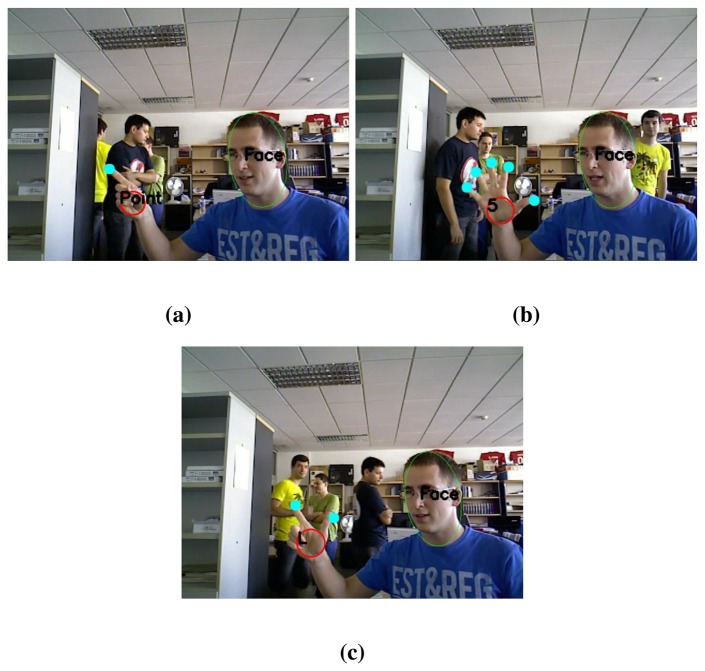
Examples of the system performance in presence of cluttered backgrounds.

Three examples with a complex and cluttered background are shown in Figure 13. The system proves to be robust when different people that are not users of the system appear in the background.

**Figure 14.**
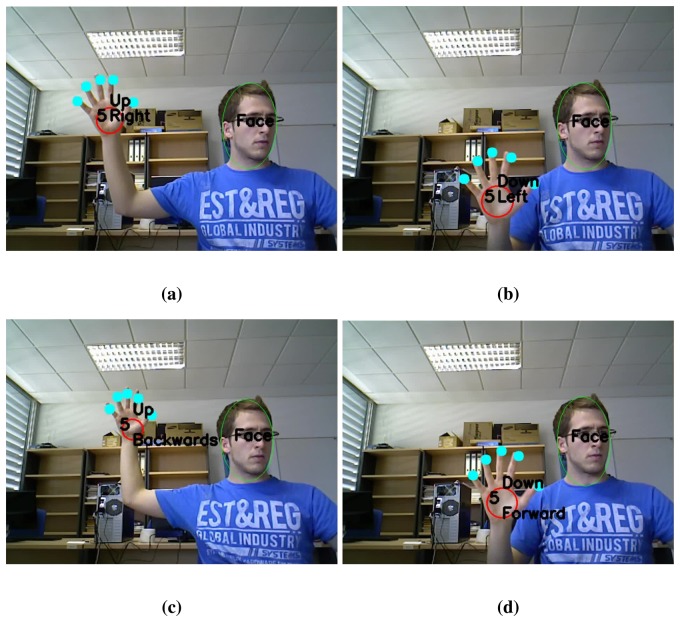
Several frames of the dynamic gesture recognition video demonstration: a) *Up* and *right* gestures; b) *Down* and *left* gestures; c) *Up* and *backwards* gestures; d) *Down* and *forward* gestures.

In order to test and present the performance of our static and dynamic gesture recognition system, a video demonstration is attached and several frames are shown in Figure 14. The system runs on an Intel Core i7 at a frame rate of 25 fps that is real time for gesture recognition applications.

## 6. Conclusions

In this paper we have proposed a new approach to hand gesture recognition combining RGB and 3-dimensional information provided by a vision and depth sensor. We have taken advantage of the depth information to address the most recurrent problem of conventional video-based skin segmentation methods, that is, to cope with cluttered backgrounds with skin colour-like objects. Subsequently, we have performed a skin colour segmentation to the foreground objects to obtain skin regions. Additionally, we have proposed a novel method to classify these skin regions including face detection. Thereby, we have found the user's hand and arm in a random indoor scene and segmented the hand from the forearm. With the information of the hand, we have used maximums of curvature and convexity defects to detect fingertips and, together with skin segments, we have classified the represented gesture. Eventually, we have performed dynamic gesture recognition in order to identify linear movements of an open hand. The experiments carried out demonstrate the accuracy of our new method, which addresses gesture recognition in a real situation without restrictions to the user for the interaction with the system. Additionally, the attached video shows the performance of the system and its real-time applicability.

## Figures and Tables

**Table 1.** Palm circle selection look-up table.

**Number of segments**		
**Circle A**	**Circle B**	**Selected Circle**
≠2	2	A
2	≠2	B
2	2	Lower Σ longitude

**Table 2.** Comparison of thresholds for face detection.

	**Habili**	**Palacios**
Orientation	±40°	±20°
Aspect Ratio	[1.40, 1.80]	[1.30, 2.43]
Relation of areas	>0.8	>0.7

**Table 3.** Results of the gesture recognition experiments.

Total number of images	90
Total number of gestures	90
Number of gestures correctly recognized (*tp*)	70
Number of gestures incorrectly recognized (*fp*)	6
Number of gestures not recognized (*fn*)	14
*Precision*	92.1
*Recall*	83.3
